# Effects of Phosphorylatable Short Peptide-Conjugated Chitosan-Mediated *IL-1Ra* and *igf-1* Gene Transfer on Articular Cartilage Defects in Rabbits

**DOI:** 10.1371/journal.pone.0112284

**Published:** 2014-11-12

**Authors:** Ronglan Zhao, Xiaoxiang Peng, Qian Li, Wei Song

**Affiliations:** Department of Medical Laboratory, Shandong Provincial Key Laboratory of Clinical Laboratory Diagnostics, Weifang Medical University, Weifang, Shandong, China; Toronto Western Hospital, Canada

## Abstract

Previously, we reported an improvement in the transfection efficiency of the plasmid DNA-chitosan (pDNA/CS) complex by the utilization of phosphorylatable short peptide-conjugated chitosan (^p^SP-CS). In this study, we investigated the effects of ^p^SP-CS-mediated gene transfection of interleukin-1 receptor antagonist protein (IL-1Ra) combined with insulin-like growth factor-1 (IGF-1) in rabbit chondrocytes and in a rabbit model of cartilage defects. pBudCE4.1-*IL-1Ra*+*igf-1,* pBudCE4.1-*IL-1Ra* and pBudCE4.1-*igf-1* were constructed and combined with ^p^SP-CS to form pDNA/^p^SP-CS complexes. These complexes were transfected into rabbit primary chondrocytes or injected into the joint cavity. Seven weeks after treatment, all rabbits were sacrificed and analyzed. High levels of *IL-1Ra* and *igf-1* expression were detected both in the cell culture supernatant and in the synovial fluid. *In vitro*, the transgenic complexes caused significant proliferation of chondrocytes, promotion of glycosaminoglycan (GAG) and collagen II synthesis, and inhibition of chondrocyte apoptosis and nitric oxide (NO) synthesis. *In vivo*, the exogenous genes resulted in increased collagen II synthesis and reduced NO and GAG concentrations in the synovial fluid; histological studies revealed that pDNA/^p^SP-CS treatment resulted in varying degrees of hyaline-like cartilage repair and Mankin score decrease. The co-expression of both genes produced greater effects than each single gene alone both *in vitro* and *in vivo*. The results suggest that ^p^SP-CS is a good candidate for use in gene therapy for the treatment of cartilage defects and that *igf-1* and *IL-1Ra* co-expression produces promising biologic effects on cartilage defects.

## Introduction

Adult articular chondrocytes have a poor intrinsic repair capacity [1], and the treatment of cartilage injury has been a problem in the field of orthopedics. With the development of molecular biology and genetic engineering techniques, gene therapy has attracted more and more attention [2]–[4]. Previously, enormous efforts have been made to develop safe and efficient gene delivery methods [5]–[7]. As a non-viral gene carrier, chitosan has been extensively studied by many researchers. DNA/chitosan (DNA/CS) nanoparticles carrying siRNA or therapeutic genes have been used for transfection of mammalian cells both *in vitro* and *in vivo* [8]–[11]. However, at present the transfection efficiency of DNA/CS microparticles is still relatively low, preventing their use as ideal gene carriers for the repair of cartilage injury. Many strategies have been employed to increase the transfection efficiency of chitosan [12]–[15]. Our research group has also been studying how to improve the transfection efficiency of chitosan [16]–[18]. Previously, we reported that phosphorylatable short peptide- (with the amino acid composition of “LLLRRRDNEY*FY*VRRLL,” containing two potentially phosphorylatable tyrosine residues) conjugated chitosan (^p^SP-CS) improved the transfection efficiency of the plasmid DNA-chitosan (pDNA/CS) complex in the 3T3, 2T3, MG63 and COS-7 cell lines [17]. Therefore, we proposed the use of ^p^SP-CS as a gene carrier in gene therapy for the treatment of cartilage defects. However, it is well known that the structure of chondrocytes is significantly different than that of the above cell lines, and the transfection efficiency of ^p^SP-CS was previously detected only *in vitro* by our team. A number of questions remain, such as how to ensure the transfection efficiency of ^p^SP-CS in chondrocytes, whether the same effects can be achieved *in vivo*, whether the administration of an intra-articular injection affects the stability of the nanoparticles, and whether ^p^SP-CS can become a reliable and efficient carrier in gene therapy for cartilage defects. Therefore, this paper attempts to use ^p^SP-CS to transport exogenous genes into chondrocytes both *in vitro* and *in vivo*, thereby improving the efficiency of exogenous gene transfection and expression.

Regarding the selection of therapeutic genes, a growing number of studies have used multi-gene combinations to treat cartilage defects [1], [3], [19], [20]. Multiple cytokines have been identified as playing important roles in the metabolism of normal cartilage [21]–[24]. A combination of antagonistic genes that inhibit extracellular matrix degradation and protective genes that promote chondrocyte-mediated biosynthesis of collagen and proteoglycan may be the most effective strategy for achieving cartilage matrix synthesis and cartilage defect repair. Prominent among these genes are interleukin-1 receptor antagonist protein (*IL-1Ra*) and insulin-like growth factor I (*igf-1*). IL-1Ra can bind the interleukin-1 (IL-1) receptor and inhibit the degradation of the extracellular matrix of articular cartilage by blocking the adverse effects of IL-1 in articular cartilage destruction [25]–[27]. IGF-1 is considered to be one of the most critical growth factors for maintaining cartilage metabolism and a stable environment [23], [28], [29]. IGF-1 plays important roles during the different stages of cartilage development, including the following: IGF-1 can stimulate glycosaminoglycan, collagen II and proteoglycan synthesis; IGF-1 can stimulate cartilage cell colony formation and cell proliferation; and IGF-1 can inhibit chondrocyte apoptosis and maintain the chondrocyte phenotype [30]–[32]. IGF-I is considered a candidate for involvement in the repair of articular cartilage, and studies have shown that it can obviously promote repair *in vitro* and in animal models [23], [28], [32]. The purpose of this research was to investigate the effects of pDNA/^p^SP-CS both *in vitro* in cultured rabbit chondrocytes and *in vivo* in an animal model of cartilage defects. In this study, *IL-1Ra* and *igf-1* were combined; the synergistic effects of these two genes were expected to gradually transform the loss of cartilage structure into tolerance and a state of slow repair, thereby achieving the goal of developing treatments for cartilage damage.

## Materials and Methods

### Animals and reagents

Chitosan (CS) was purchased from Sigma (Sigma–Aldrich Co., USA) with a molecular mass  = 60000–100000 Da (degrees of deacetylation up to approximately 85%). DMEM/F12 (Dulbecco's Modified Eagle Medium/F12) cell culture medium was purchased from Gibco (USA), and collagenase type II was purchased from Sigma (Sigma-Aldrich Co, USA). Three-month-old New Zealand white rabbits (2.0–2.5 kg) were obtained from Shandong Lukang Pharmaceutical Limited by Share Ltd. (Shandong, China). Protocols involving animals used in this study were approved by the Institutional Animal Care and Use Committee of Weifang Medical University.

We also purchased the following kits: human IGF-1 enzyme linked immunosorbent assay (ELISA) kit (U.S. R & D); human IL-1RA enzyme linked immunosorbent assay kit (U.S. eBioscience); rabbit glycosaminoglycan (GAG) enzyme linked immunosorbent assay kit (Nanjing Sen Beijia biotechnology Co., Ltd. China); Annexin V-FITC apoptosis detection kit and nitrate reductase kit of NO (NanJing JianCheng Bioengineering Institute, China); TRIzol total RNA extraction reagent and quantitative real-time PCR detection kit (Takara, Shiga, Japan); M-MLV reverse transcriptase (Invitrogen, Carlsbad, CA, USA); and KOD -Plus- Ver polymerase (TOYOBO, Tokyo, Japan).

### Construction of pBudCE4.1 vectors

Using PCR, we obtained the total sequence of *igf-1* cDNA from the pTRACER-c*igf-1* (previously constructed and saved by our team). The forward primer 5′-GGGGTACCATGGGAAAAATCAGCAGTCTTC-3′ contains the restriction endonuclease site of *Kpn*I, and the reverse primer 5′-CCGCTCGAG CTAAGCTGACTTGGCAG-3′ contains an *Xho*I site. Normal human peripheral blood lymphocytes (The participant provided written informed consent, the study was carried out in accordance with the Declaration of Helsinki and was approved by the Institutional Review Board of Weifang Medical University) were isolated and activated by phytohaemagglutinin (PHA) for 24 h; then the cells were collected, and total RNA was extracted using TRIzol reagent. Subsequently, the total RNA was used for reverse transcription to produce cDNA. Using the “total cDNA” described above as a template for PCR, we obtained the total sequence of *IL-1Ra* cDNA. The forward primer 5′-CCCAAGCTTATGGAAATCTGCAGAGGCCT-3′ contains the restriction endonuclease site of *Hin*dIII, and the reverse primer 5′-CGCGGATCCCTACTCGTCCTCCTGGAAGTA-3′ contains a *Bam*HI site. The *Kpn*I*-Xho*I and *Hin*dIII-*Bam*HI PCR product fragments were subcloned into pBudCE4.1 at the corresponding sites, individually or together. The transcription of *igf-1* and *IL-1Ra* was driven by the cytomegalovirus (CMV) promoter and elongation factor 1α-subunit (EF-1α), respectively. The constructed co-expression plasmid pBudCE4.1-*IL-1Ra*+*igf-1* and the single-expression plasmids pBudCE4.1-*IL-1Ra* and pBudCE4.1-*igf-1* were confirmed via sequence analysis.

### Preparation of pDNA/^p^SP-CS complexes

The phosphorylatable short peptide (“LLLRRRDNEY*FY*VRRLL”) was conjugated to chitosan to form ^p^SP-CS as previously described [17]. pBudCE4.1-*IL-1Ra*+*igf-1,* pBudCE4.1-*IL-1Ra,* pBudCE4.1-*igf-1* and the empty plasmid pBudCE4.1 were extracted, and the pDNA/^p^SP-CS complex was prepared as follows: Plasmid DNA was dissolved in TE buffer to a final concentration of 1 µg/µL. The above-described ^p^SP-CS was dissolved in acetic acid (pH 5.4) to a final concentration of 1 µg/µL. The recombinant plasmid and ^p^SP-CS were mixed in weight ratios of 1∶0.25, 1∶0.5, 1∶0.75, 1∶1, 1∶1.5 or 1∶2, vortexed immediately and incubated at room temperature for 30 min to allow the complexes to form. The pDNA/^p^SP-CS complexes were subjected to agarose gel electrophoresis, and the formation of the complexes was verified by gel retardation. The pDNA/^p^SP-CS complexes (pBudCE4.1-*IL-1Ra*+*igf-1*/^p^SP-CS, pBudCE4.1-*IL-1Ra*/^p^SP-CS, pBudCE4.1-*igf-1*/^p^SP-CS and pBudCE4.1/^p^SP-CS) were prepared by the method described above using a pDNA:^p^SP-CS weight ratio of 1∶1.5 in the follow-up experiments. EGFP reporter gene plasmid pEGFP-C1 was extracted and mixed respectively with ^p^SP-CS and CS on the weight ratio of 1∶1.5 to form the pEGFP/^p^SP-CS complex and pEGFP/CS complex.

### *In vitro* experiment

#### Chondrocyte cell isolation and culture transfection

One-week-old New Zealand white rabbit were obtained from Shandong Lukang Pharmaceutical Limited by Share Ltd (Shandong, China). Surgical procedures were performed and the rabbits were sacrificed under inhalation anesthesia induced by diethyl ether. Rabbit articular cartilage was separated from the connective tissue and underlying bone. First, the samples were digested for 1 h in 0.25% trypsin and centrifuged at 1200 r/min for 5 min. The supernatant was removed, and the samples were washed with PBS. Then, the samples were digested for 17–20 h in 0.02% collagenase type II in Dulbecco's Modified Eagle's Medium/F12 (DMEM/F12) medium. The chondrocytes were placed in primary monolayer cultures in DMEM/F12 medium with 10% FBS at 37°C with 5% CO_2_. The second-generation chondrocytes were used for the following experiments.

Chondrocytes were seeded at a density of 1×10^4^/mL and 1×10^6^/mL on 96-well and 6-well microplates in DMEM/F12 medium containing 10% FBS. Cells were cultured at 37°C with 5% CO_2_. When the cells had grown to half confluence, the pDNA/^p^SP-CS complexes were added to the 96-well and 6-well plates to achieve DNA quantities of 0.25 µg/well and 4 µg/well. At 24 h of transfection, cells were treated with IL-1β (10 ng/mL) [33], [34]. The chondrocytes were divided into five groups: (1) chondrocytes without transfection, (2) chondrocytes transfected with pBudCE4.1/^p^SP-CS, (3) chondrocytes transfected with pBudCE4.1-*IL-1Ra*/^p^SP-CS, (4) chondrocytes transfected with pBudCE4.1-*igf-1*/^p^SP-CS, and (5) chondrocytes transfected with pBudCE4.1-*IL-1Ra+igf-1*/^p^SP-CS. The chondrocytes in the 96-well microplates were used to detect cell proliferation. The cells in the 6-well microplates were used to detect cell apoptosis; collagen II expression; and the concentrations of GAG, NO, exogenous IL-1Ra protein, and IGF-1 protein in cell supernatant. When detecting exogenous IL-1Ra protein and IGF-1 protein in cell supernatant, DNA/CS complexes were added to the 6-well at same time, established as control.

When the second-generation chondrocytes had grown to half confluence, the pEGFP/^p^SP-CS complex and pEGFP/CS complex were added respectively to the 6-well plates to achieve DNA quantities of 4 µg/well, non-transfected cells cultured as control. At 48 h of transfection, GFP was observed under fluorescent microscope and the transfection efficiency was detected by flow cytometry.

#### IGF-1, IL-1Ra, GAG and NO concentrations in cell supernatants

Seven days after transfection, cell supernatants were collected and analyzed to determine the concentrations of IL-1Ra, IGF-1 and GAG by ELISA kits according to the manufacturer's instructions. NO accumulation in the cell supernatant was measured with a nitrate reductase kit according to the manufacturer's protocols.

#### Assay of chondrocytes proliferation and apoptosis

An MTT assay was employed to detect cell proliferation. Chondrocytes were seeded into 96-well plates and transfected as described above. Then, 48 h after the transfection, sterile filtered MTT solution (20 µL; 5 mg/mL) was added to each well, then incubated at 37°C with 5% CO_2_ for 4 h. The formazan crystals were dissolved in dimethylsulfoxide (200 µL/well), and the absorbance at 570 nm was measured using a microplate reader (BIO-RAD, USA). Each group experiment was repeated six times.

Apoptosis of the chondrocytes was detected using flow cytometry (BD, USA) with Annexin V-FITC labeling. The cells were seeded into 6-well plates and transfected as described above. Seven days after the transfection, the cells were collected and detected using an Annexin V-FITC apoptosis detection kit according to the manufacturer's protocols.

#### RT-qPCR of collagen II mRNA

Chondrocytes were seeded into 6-well plates and transfected as described above. The cells were collected 7 d after the transfection, and the total RNA was extracted using TRIzol reagent. The mRNA (2 µg) was reverse transcribed into total cDNA in a 20 µL reaction mixture. The expression levels of the collagen II mRNA were detected via quantitative real-time RT-PCR (RT-qPCR) in iQ5TM (BIO-RAD, USA), using the β-2-microglobulin (B2M) gene as a reference gene [35]. Two pairs of primers were used as follows: collagen II sense 5′-ATggCggCTTCCACTTCAg-3′ and antisense 5′-CggTggCTTCATCCAggTAg-3′; and B2M sense 5′-AACgTggAACAgTCAgACC-3′ and antisense 5′-AgTAATCTCgATCCCATTTC-3′. A total reaction volume of 20 µL was comprised of 10 µL of 2×SYBR Green Mix, 1 µL of primer mix (200 nM), 2 µL of cDNA and 7 µL of sterile distilled water. The qPCR conditions were as follows: 95°C for 30 sec, followed by 35 cycles at 95°C for 5 sec, 62°C for 15 sec and 72°C for 20 sec. The PCR was followed immediately by a melting analysis. Each qPCR assay was run with triplicate technical samples. The CT values of all of the genes from different samples were gathered, the raw data were calibrated to the *B2M* reference gene, the mRNA of each sample was normalized, and the relative expression level of collagen II was represented as 2^-ΔCT^.

### *In vivo* experiment

#### Injury to articular cartilage

Thirty New Zealand White rabbits (2.0–2.5 kg) were randomly divided into five major groups: the joint cavity only was opened and sutured in the first group (sham operated group), and artificial cartilage full-thickness defects were induced in groups 2 to 5, as previously described [35]. Then, the experimental rabbits were housed in individual cages with standard food and water *ad libitum* under normal conditions. All procedures involving animals were approved by the Animal Care and Use Committee of China. Intramuscular injection of 400,000 U of penicillin and disinfection of the skin wounds were performed for 5 days after surgery.

Sterile saline, pBudCE4.1/^p^SP-CS, pBudCE4.1-*IL-1Ra*/^p^SP-CS, pBudCE4.1-*igf-1*/^p^SP-CS and pBudCE4.1-*IL-1Ra*+*igf-1*/^p^SP-CS were injected into the joint cavities of groups 1 through 5, respectively. Seven days after the surgery, intra-articular injections were administered twice a week for 7 weeks. The amount of plasmid DNA in the each of the intervention groups was 15 µg (each time), and the pDNA/^p^SP-CS complexes were dissolved in saline to adjust the volume to 0.5 mL. The sham control group was injected with 0.5 mL 0.9% (w/v) physiological saline. Seven weeks after the intra-articular injection, the rabbits were sacrificed under general anesthesia. The knee joint cavity was lavaged using sterile saline (repeated 3 times) through the patellar tendon. After centrifugation, the joint cavity lavage fluid (synovial fluid) was used to analyze the concentrations of IL-1Ra, IGF-1, GAG and NO. RNA was isolated from one half of each group and used for RT-qPCR (n = 6). The remaining half of each group was subjected to histological evaluation (n = 6).

#### IGF-1, IL-1Ra, GAG and NO concentrations in synovial fluid

The concentrations of IL-1Ra, IGF-1 and GAG in the synovial fluid were detected using ELISA kits according to the manufacturer's instructions. NO accumulation in synovial fluid was measured with a nitrate reductase kit according to the manufacturer's protocols.

#### RT-qPCR of collagen II mRNA

The cartilage surrounding the defect was carefully trimmed off and immediately put into TRIzol reagent. The total RNA was extracted using TRIzol reagent and was reverse transcribed into cDNA as described above. The expression levels of collagen II mRNA were detected via quantitative real-time RT-PCR (RT-qPCR) as described above.

#### Histological examination of articular cartilage injury and repair

Following the dissection, the region encompassing the whole defect, including some hard bone tissue and the surrounding cartilage, was fixed in 10% buffered formalin for 24 h. Then, the specimens were decalcified in 0.5% EDTA solution and embedded in paraffin. Four-micrometer sagittal sections of the defect area were collected and stained with hematoxylin and eosin (HE), Safranin O/fast green staining and immunohistochemistry according to a standard protocol. The structure of articular cartilage tissue was observed by optical microscope, and the quantification of cartilage damage was graded histologically according to Mankin et al [36].

### Statistical tests

All measurement data are expressed as the mean ± the standard deviation and were analyzed by SPSS 17.0. The single-factor analysis of variance was used for the multi-group comparisons. Each result was compared using the Student-Newman-Keuls test. A value of P<0.05 was considered significant.

## Results

### Agarose gel electrophoresis of pDNA/^p^SP-CS complexes

**Figure 1:**
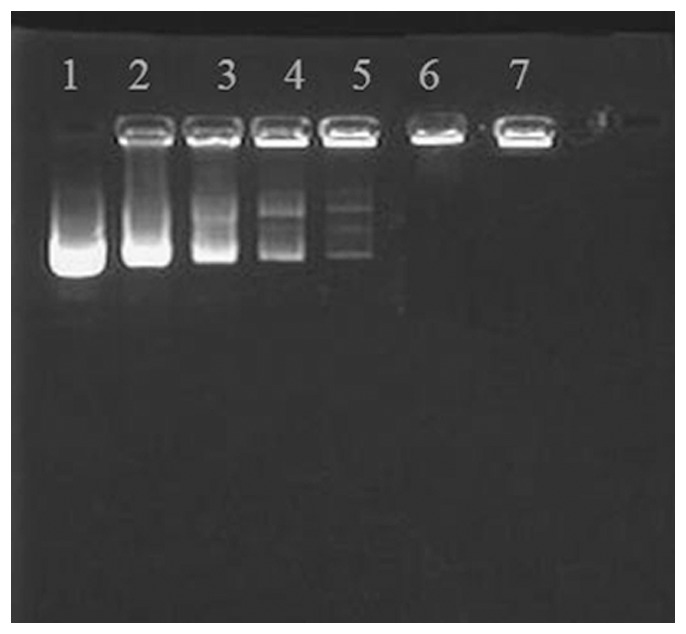
Agarose gel electrophoresis of the pDNA/^p^SP-CS complexes. Lane 1: free plasmid DNA; lane 2–7: pDNA:^p^SP-CS (w/w)  = 1:0.25; 1:0.5; 1:0.75; 1:1; 1:1.5; 1:2. When pDNA:^p^SP-CS reached 1:1.5, these complexes lost their mobility in the gel.

The recombinant plasmid and ^p^SP-CS were mixed in weight ratios of 1∶0.25, 1∶0.5, 1∶0.75, 1∶1, 1∶1.5, and 1∶2. When the pDNA/^p^SP-CS ratio was at or beyond 1∶1.5, the pDNA/^p^SP-CS complexes exhibited no migration during electrophoresis, having lost the ability to move toward the positive electrode (Fig. 1).

### Comparing IGF-1 and IL-1Ra concentrations in the cell supernatant between DNA/CS and DNA/^p^SP-CS transfected group

From Table 1 and 2, it was seen that the ^p^SP conjugation increases the expression of *igf-1* and *IL-1Ra* gene markedly.

**Table 1:** Human *igf-1* gene expression levels in cell supernatants determined by ELISA, n = 3(ng/mL).

	control group	pBudCE4.1	pBudCE4.1-*igf-1*	pBudCE4.1-*IL-1Ra*	pBudCE4.1-*IL-1Ra*+*igf-1*
DNA/CS	0.040±0.004	0.042±0.005	0.594± 0.042^a^	0.041±0.007	0.595±0.025 a
DNA/^p^SP-CS	0.050±0.004	0.053±0.005	1.161± 0.089*^b^*	0.051±0.007	1.164±0.023 *^b^*

Between DNA/CS and DNA/^p^SP-CS transfected groups, there are significant difference between *^a^* and *^b^* marked results (*^a^* vs *^b^*, *P*<0.05);

a and *^b^* marked results have significant difference with other unmarked ones (*p*<0.05).

**Table 2:** Human *IL-1Ra* gene expression levels in cell supernatants determined by ELISA, n = 3(pg/mL).

	control group	pBudCE4.1	pBudCE4.1-*igf-1*	pBudCE4.1-*IL-1Ra*	pBudCE4.1-*IL-1Ra*+*igf-1*
DNA/CS	74.908±6.123	75.923±6.758	83.342± 5.783	657.689±19.253 a	662.610±22.789 a
DNA/^p^SP-CS	80.802±6.706	82.212±6.745	91.132± 4.874	1288.612± 15.158 *^b^*	1298.247±15.510 *^b^*

Between DNA/CS and DNA/^p^SP-CS transfected groups, there are significant difference between *^a^* and *^b^* marked results (*^a^* vs *^b^*, *P*<0.05);

a and *^b^* marked results have significant difference with other unmarked ones (*p*<0.05).

### Comparing the transfection efficiency between pEGFP/^p^SP-CS and pEGFP/CS transfected group

**Figure 2:**
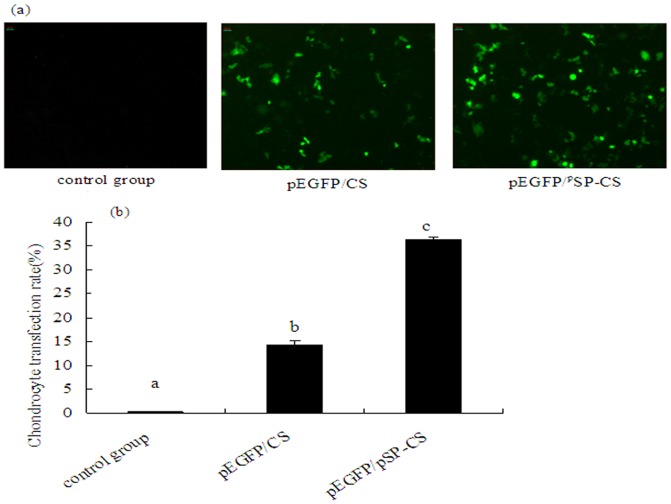
The transfection efficiency of pEGFP/^p^SP-CS and pEGFP/CS in rabbit primary cultured chondrocytes. Chondrocytes were transfected with pEGFP/^p^SP-CS and pEGFP/CS for 48 h, then GFP was observed under fluorescent microscope (×100, 2A) and the transfection efficiency was detected by flow cytometry (2B). The data are shown as the mean ±S.D. a, b, and c show the transfection efficiency in each group compared with p<0.01 (n = 6, 2B).

In the in *vitro* experiments, at 48h of transfection, fluorescent microscope and flow cytometry showed that the transfection efficiency of pEGFP/^p^SP-CS was significantly higher than that of pEGFP/CS, the results were shown in Figure. 2.

### IGF-1 and IL-1Ra concentrations in the cell supernatant and synovial fluid

#### IGF-1 analysis

**Figure 3:**
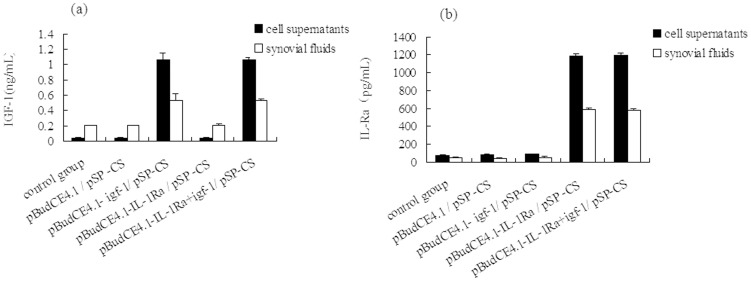
^p^SP-CS-mediated human IGF-1 and IL-1Ra gene expression levels in cell supernatants and synovial fluid from rabbit knees. (**A**) The concentration of IGF-1 in the cell supernatant and synovial fluid was analyzed by ELISA. The data are shown as the mean ±S.D. A similarly high level of IGF-1 expression was detected in the pBudCE4.1-*IL-1R*a+*igf-1/* ^p^SP-CS and pBudCE4.1-*igf-1/* ^p^SP-CS transfection groups. No significant difference was observed among the pBudCE4.1-*IL-1Ra/* ^p^SP-CS, pBudCE4.1*/* ^p^SP-CS and control groups. (**B**) The concentration of IL-1Ra in the cell supernatant and synovial fluid was analyzed by ELISA. The data are shown as the mean ± S.D. A similarly high level of IL-1Ra expression was detected in the pBudCE4.1-*IL-1R*a+*igf-1/* ^p^SP-CS and pBudCE4.1-*IL-1Ra/* ^p^SP-CS transfection groups. No significant difference was observed among the pBudCE4.1-*igf-1/* ^p^SP-CS, pBudCE4.1*/* ^p^SP-CS and control groups.

In the *in vitro* and *in vivo* experiments, the pBudCE4.1-*igf-1*/^p^SP-CS-transfected group and the co-expression plasmid pBudCE4.1-*IL-1Ra*+*igf-1*/^p^SP-CS-transfected group showed significantly higher IGF-1 expression levels than the non-transfected control group (p<0.05), and no significant difference between the two transfected groups was observed; in the pBudCE4.1/^p^SP-CS-transfected and the pBudCE4.1-*IL-1Ra*/^p^SP-CS-transfected groups, IGF-1 expression levels were not significantly different from the control group (p>0.05) (Fig. 3A).

#### IL-1Ra analysis

In the *in vitro* and *in vivo* experiments, the pBudCE4.1-*IL-1Ra*/^p^SP-CS-transfected group and the co-expression plasmid pBudCE4.1-*IL-1Ra*+*igf-1*/^p^SP-CS-transfected group showed significantly higher IL-1Ra expression levels than the non-transfected control group (p<0.05), and no significant difference between the two transfected groups was observed; in the pBudCE4.1/^p^SP-CS-transfected and the pBudCE4.1-*igf-1*/^p^SP-CS-transfected groups, IL-1Ra expression levels were not significantly different from the control group (p>0.05) (Fig. 3B).

### GAG concentrations in cell supernatant and synovial fluid

#### In cell supernatant

**Figure 4:**
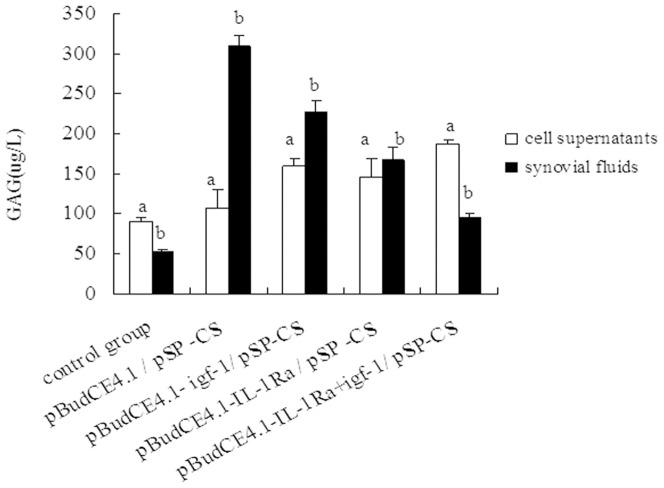
Concentration of GAG in cell supernatant and synovial fluid. The concentration of GAG in cell supernatant and synovial fluid was measured by ELISA. The data are shown as the mean ±S.D. a shows GAG contents in cell supernatant of each group compared with p<0.05; b shows GAG in synovial fluid from the rabbit knees of each group compared with p<0.05.

Compared with the control group, the groups transfected with pBudCE4.1/^p^SP-CS, pBudCE4.1-*IL-Ra*/^p^SP-CS, pBudCE4.1-*igf-1*/^p^SP-CS or pBudCE4.1-*IL-1Ra*+*igf-1*/^p^SP-CS showed increased amounts of GAG (p<0.05) (Fig. 4).

#### In synovial fluid

Compared with the control group, all of the surgical groups showed elevated GAG levels. The highest levels of GAG were detected in the pBudCE4.1/^p^SP-CS transfection group, followed by the pBudCE4.1-*igf-1*/^p^SP-CS, pBudCE4.1-*IL-1Ra*/^p^SP-CS and pBudCE4.1-*IL-1Ra*+*igf-1*/^p^SP-CS groups (p<0.05) (Fig. 4).

### NO levels in cell supernatant and synovial fluid

#### In cell supernatant

**Figure 5:**
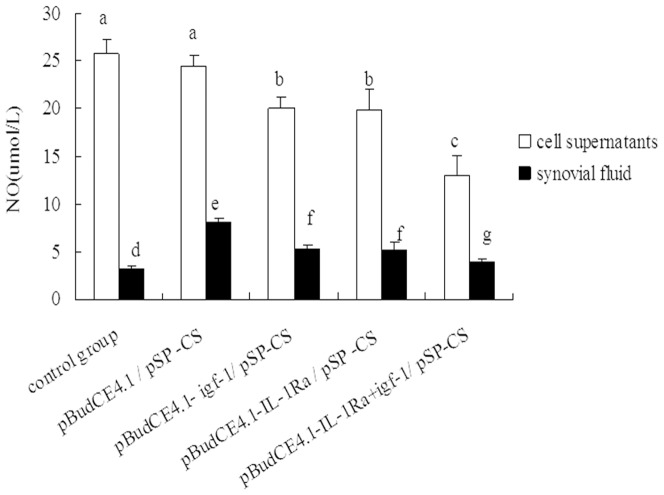
The levels of nitric oxide (NO) in cell supernatant and synovial fluid. The NO contents of the cell supernatant and synovial fluid were measured by a nitrate reductase assay. The data are shown as the mean ±S.D. a, b and c show the NO contents in the cell supernatant from each group compared with p<0.05; d, e, f and g show the NO contents of the synovial fluid from the rabbit knees of each group compared with p<0.05.

The lowest levels of NO were detected in the pBudCE4.1-*IL-1Ra*+*igf-1/* ^p^SP-CS transfection group. There were no significant differences in NO levels between the control group and the pBudCE4.1/^p^SP-CS transfection group, but these levels were found to be higher than that of the other groups. No significant difference was observed between the pBudCE4.1-*igf-1*/^p^SP-CS and pBudCE4.1-*IL-1Ra*/^p^SP-CS transfection groups (Fig. 5).

#### In synovial fluid

The highest levels of NO were detected in the pBudCE4.1/^p^SP-CS-transfected group. There were no significant differences in NO levels between the control group and the pBudCE4.1-*IL-1Ra*+*igf-1*/^p^SP-CS transfection group, but these levels were found to be lower than that of the other groups. No significant difference was observed between the pBudCE4.1-*igf-1*/^p^SP-CS and pBudCE4.1-*IL-1Ra*/^p^SP-CS transfection groups (Fig. 5).

### Assay of chondrocyte proliferation and apoptosis

#### Chondrocyte proliferation assay

**Figure 6:**
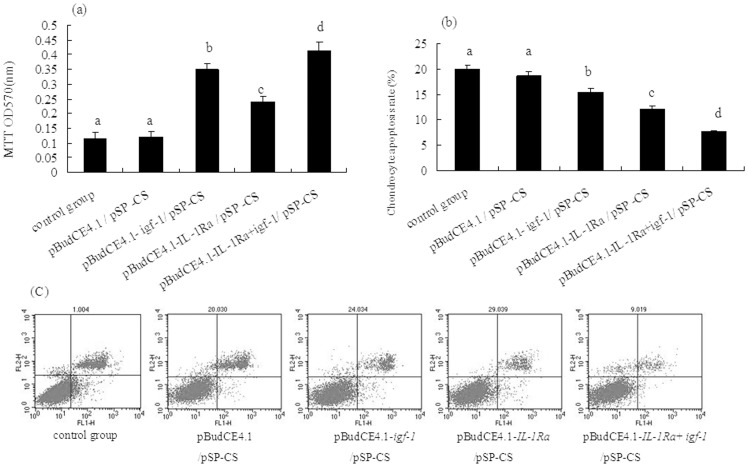
Chondrocyte proliferation and apoptosis analysis. (A) Rabbit chondrocyte proliferation was detected by the MTT method *in vitro*. The data are shown as the mean ±S.D. a, b, c and d show cell proliferation in each group compared with p<0.05. (B-C) Rabbit chondrocyte apoptosis was detected by an Annexin V-FITC assay. The total number of apoptotic cells was determined by calculating the sum of the early apoptotic cells (Annexin V-FITC +/PI-) and the late apoptotic cells (Annexin V-FITC+/PI+) detected by flow cytometry (6C); the data are shown as the mean ±S.D. a, b, c and d show the chondrocyte apoptosis rate in each group compared with p<0.05 (6B).

Chondrocyte proliferation was shown to be significantly increased in the group transfected with the co-expression plasmid pBudCE4.1-*IL-1Ra*+*igf-1*/^p^SP-CS, followed by the pBudCE4.1-*igf-1*/^p^SP-CS, pBudCE4.1-*IL-1Ra*/^p^SP-CS, pBudCE4.1/^p^SP-CS and control groups. However, there was no significant difference in chondrocyte proliferation between the pBudCE4.1/^p^SP-CS group and the control group (Fig. 6A).

#### Chondrocyte apoptosis assay

To examine the impact of the exogenous genes on chondrocyte apoptosis, we evaluated cell apoptosis using an Annexin V-FITC assay. The total number of apoptotic cells was determined as the sum of the early apoptotic cells (Annexin V-FITC+/PI-) and the late apoptotic cells (Annexin V-FITC+/PI+). As shown in Fig 6B and Fig 6C, chondrocyte apoptosis was significantly reduced in the group transfected with the co-expression plasmid pBudCE4.1-*IL-1Ra*+*igf-1*/^p^SP-CS, followed by the pBudCE4.1-*IL-1Ra*/^p^SP-CS, pBudCE4.1-*igf-1*/^p^SP-CS, pBudCE4.1/^p^SP-CS and control groups. However, there was no significant difference in chondrocyte apoptosis between the pBudCE4.1/^p^SP-CS-transfected group and the control group.

### Quantitative collagen II expression in chondrocytes and cartilage

**Figure 7:**
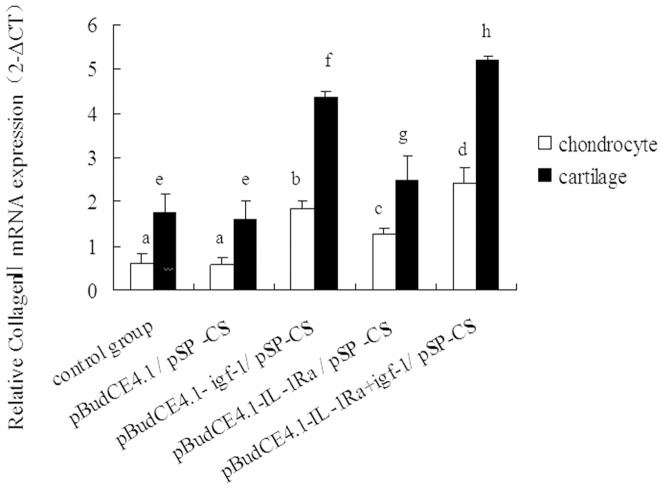
The relative expression of *collagen II* mRNA in different groups was determined by RT-qPCR. The data are shown as the mean ±S.D. The raw *collagen II* mRNA expression data for each group were calibrated to the *B2M* reference gene, and the relative expression level of *collagen II* was represented as 2^−△CT^. a, b, c and d show the relative expression of *collagen II* mRNA in each cultured chondrocyte group compared with p<0.05; e, f, g and h show the relative expression of *collagen II* mRNA in cartilage tissue from rabbit knees compared with p<0.05.

We determined the relative expression levels of collagen II in the cultured chondrocytes and cartilage tissue for each of the different transfection groups. Compared with the control and pBudCE4.1/^p^SP-CS-transfected groups, the pBudCE4.1-*IL-1Ra*/^p^SP-CS, pBudCE4.1-*igf-1*/^p^SP-CS, and pBudCE4.1-*IL-1Ra*+*igf-1/* ^p^SP-CS transfection groups showed a gradual but significant increase in collagen II expression (p<0.05). However, no significant difference in collagen II expression was detected between the control group and the pBudCE4.1/^p^SP-CS group (Fig. 7).

### Histological examination of articular cartilage

#### HE staining

**Figure 8:**
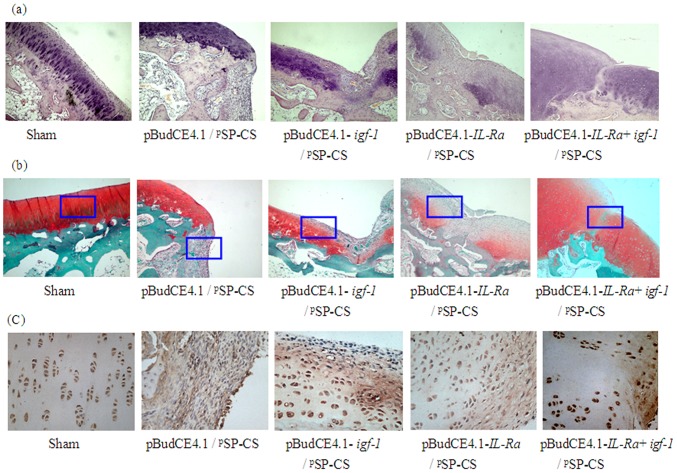
The histological analysis of articular cartilage. Seven weeks after ^p^SP-CS/DNA was injected into the joint cavity, the rabbits were sacrificed, four-micrometer sagittal sections of the medial femoral condyles were stained with HE (A, ×100), Safranin O/fast green (B, ×100) and collagen II (C, ×400, from the enlarged portion of the blue box in figure B). In each transgenic group, cartilage defects were filled with different degrees of nascent cartilage, and this cartilage functioned similarly to the normal cartilage. The co-transfected group of pBudCE4.1-*IL-Ra*+*igf-1/* ^p^SP-CS showed greater effects than the other two single-gene-transfected groups.

In the cartilage of the undamaged sham group, the articular cartilage had a smooth and continuous surface, the cartilage matrix was uniformly stained, and the four-layer structure of the articular cartilage was clear. In the pBudCE4.1/^p^SP-CS group, the defect was found to be partially filled with inflammatory cells and fibrous tissue; different levels of cartilage repair appeared in the pBudCE4.1-*igf-1*/^p^SP-CS, pBudCE4.1-*IL-1Ra*/^p^SP-CS, and pBudCE4.1-*IL-1Ra*+*igf-1*/^p^SP-CS groups. The most complete repair appeared in the co-expression plasmid pBudCE4.1-*IL-1Ra*+*igf-1/* ^p^SP-CS-transfected group; in this group, the defects were completely filled with nascent cartilage (Fig. 8A).

#### Safranin O/fast green staining

In the sham group, the intact articular cartilage had a smooth surface, normal structural organization and uniform Safranin O/fast green staining; Safranin O staining was negative in the defect of the pBudCE4.1/^p^SP-CS group. Different intensities of Safranin O staining appeared in the defects of the pBudCE4.1-*igf-1*/^p^SP-CS, pBudCE4.1-*IL-1R*a/^p^SP-CS, and pBudCE4.1-*IL-1Ra*+ *igf-1/* ^p^SP-CS groups. The most intense staining appeared in the pBudCE4.1-*IL-1Ra*+*igf-1/* ^p^SP-CS group; the defect was completely filled with nascent cartilage and showed intense staining by Safranin O (Fig. 8B). Mankin scores were shown in Table 3.

**Table 3:** Mankin scores of articular cartilage specimens (point).

Group	N	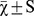
Sham	6	0.330±0.516 *^a^*
pBudCE4.1*/* ^p^SP-CS	6	11.830±1.169 *^b^*
pBudCE4.1-*igf-1/* ^p^SP-CS	6	7.170±0.408 *^c^*
pBudCE4.1-*IL-1Ra/* ^p^SP-CS	6	8.500±0.548 *^d^*
pBudCE4.1-*IL-1R*a+*igf-1/* ^p^SP-CS	6	4.830±0.408 *^e^*

*a*, *b, c, d* and *e* show the Mankin scores of articular cartilage specimens from each group compared with p<0.05.

#### Immunohistochemistry

Chondrocytes were observed in the middle of the main layer in the sham group and were found to contain a large amount of collagen II protein. The collagen II protein was immunologically detected as dark brown staining in the extracellular matrix. In the defect of the pBudCE4.1/^p^SP-CS group, inflammatory cells and fibrous tissue produced a slight, non-specific coloration. In the defects of the pBudCE4.1-*igf-1*/^p^SP-CS, pBudCE4.1-*IL-1Ra*/^p^SP-CS, and pBudCE4.1-*IL-1Ra*+*igf-1*/^p^SP-CS groups, a large amount of collagen II protein was detected in the matrix of the new cartilage, and the chondrocyte morphology and coloration in the pBudCE4.1-*IL-1Ra*+*igf-1*/^p^SP-CS group is close to that of the sham group (Fig. 8C).

## Discussion

The identification of an efficient and safe gene transport carrier is very important for the development of gene therapy to repair cartilage defects. As a non-viral gene carrier, chitosan is a very promising candidate for use in gene therapy. Due to its good biocompatible properties, chitosan can cross-link with collagen macromolecules [37], [38]. In damaged cartilage, the cartilage cells and collagen in the defect area are exposed, which allows chitosan nanoparticles localized and expressed exogenous proteins in the defects and produce a therapeutic effect. Our previous studies are consistent with this finding; our results showed that the use of unmodified chitosan as a gene transfer vector loaded with the human *igf-1* gene and delivered *via* direct intra-articular injection can enhance the formation of mesochondrium by upregulating collagen II and aggrecan expression to some extent [39]. However, the relatively low transfection efficiency of chitosan has limited its further application in the transgenic treatment of cartilage injury. Previously, we had reported improved transfection efficiency of the plasmid DNA-chitosan (pDNA/CS) complex via enhanced intracellular unpacking of the exogene through utilization of phosphorylatable short peptide-conjugated chitosan (^p^SP-CS) in the 3T3, 2T3, MG63 and COS-7 cell lines [17]. The above results led us to propose the use of ^p^SP-CS as a gene transfer vector to mediate gene transfection in rabbit chondrocytes and in a rabbit model of cartilage defects. We hypothesized that after ^p^SP was phosphorylated, pDNA would be released from the pDNA/^p^SP-CS complex, and the exogenous gene would be highly expressed in chondrocytes, contributing to the repair of the cartilage defects.

In this study, when the ratio of pSP-CS:pDNA (W/W) was at or beyond 1.5∶1, the negative charge of the pDNA was completely shielded. We first confirmed that ^p^SP-CS can also improve the transfection efficiency of pEGFP-C1 in chondrocytes, by fluorescent microscope and flow cytometry. Then we successfully prepared four types of nanoparticles: pBudCE4.1-*IL-1Ra*+*igf-*1/^p^SP-CS, pBudCE4.1-*IL-1Ra*/^p^SP-CS, pBudCE4.1-*igf-1*/^p^SP-CS and pBudCE4.1/^p^SP-CS. pBudCE4.1. A bicistronic expression vector with a human cytomegalovirus (CMV) promoter and human elongation factor 1α-subunit (EF-1α) promoter allows for the constitutive, independent and simultaneous expression of two genes in mammalian cell lines and has been widely used to eliminate variable expression of two genes in the same mammalian cells [40]. When studying both IGF-1 and IL-1Ra, the use of the pBudCE4.1 co-expression plasmid can greatly reduce the variability caused by experimental inconsistencies in the gene transfection efficiency. In both the *in vitro* and *in vivo* experiments in this study, the ELISA results show that the introduction of the *IL-1Ra*+*igf-1* gene by the co-expression plasmid pBudCE4.1-*IL-1R*a+*igf-1* produces similar expression levels of IL-1Ra and IGF-1 as those produced by the pBudCE4.1-*IL-1Ra* and pBudCE4.1-*igf-1* single-gene transfection groups, respectively. For *in vitro igf-1* and *IL-1Ra* gene expression assay, shown in Table 1,2, compared with unconjugated CS, ^p^SP conjugation could significantly increase transfection effciency of the DNA/^p^SP-CS, as much more IL-1Ra and IGF-1 could be detected. These results suggest that ^P^SP-CS can efficiently transport the exogenous genes *IL-1Ra* and *igf-1* into chondrocytes. In addition, the exogenous IL-1Ra and IGF-1 can be synthesized and secreted by transfected chondrocytes and exposed chondrocytes in cartilage *in vivo*.

Because IGF-1 can promote chondrocyte proliferation and extracellular matrix synthesis, it has been extensively used in the repair of cartilage injury and has produced encouraging results [41], [42]. However, a large number of studies have shown that if IGF-1 alone is used, its effectiveness is limited to some extent and would be greatly improved if combined with other genes, such as bFGF [31], FGF-2 [1], [3], BMP-2, BMP-7 [3] and IL-1Ra [41]–[43]. In this study, IL-1Ra was selected for co-expression with IGF-1. By binding the IL-1 receptor (IL-1R), IL-1Ra can block the binding of IL-1, which is a major stress and inflammation cytokine that prevents the repair of damaged cartilage [41]. In human patients, studies have shown that the more serious cases of articular cartilage injury are often accompanied by a more significant decrease of IL-1Ra [44], [45]. *In vivo* IL-1Ra gene transfer promotes the repair of cartilage defects in rabbits [26]. In this study, the results demonstrated that introduction of IGF-1 and IL-1Ra by ^p^SP-CS transfection had a positive effect both on cultured rabbit chondrocytes and on cartilage repair in a rabbit cartilage defect model. The *in vitro* results showed that IGF-1 and IL-1Ra not only promoted rabbit chondrocyte proliferation and the synthesis of GAG and collagen II, but they also inhibited apoptosis and NO synthesis in chondrocytes. In *in vivo* experiments, IGF-1 and IL-1Ra reduced the concentrations of GAG and NO in the synovial fluid. GAG and collagen II are the major extracellular matrix (ECM) components of cartilage. *In vitro*, chondrocyte redifferentiation was supported by the production of glycosaminoglycan (GAG), and the biosynthesis of collagen II [46]; therefore, changes in GAG and collagen II reflect the level of anabolism of cartilage matrix. When cartilage is damaged *in vivo*, the GAG concentration in the synovial fluid begins to rise due to the degradation of the cartilage matrix [47], [48]; changes in the GAG concentration in the synovial fluid reflect the level of catabolism of the cartilage matrix. Under a variety of pathological conditions, such as cartilage ischemia, cytokine secretion, injury and other stimulation, NO expression and regulation are significantly higher than normal. Studies have shown that high concentrations of NO can significantly inhibit chondrocyte proliferation and induce chondrocyte apoptosis [49], [50]. This situation results in a reduced ability to repair cartilage, thus increasing cartilage damage and accelerating cartilage degeneration. Our *in vitro* results show that by inhibiting NO production, IGF-1 and IL-1Ra can promote the anabolism of the cartilage matrix, further stimulate cell proliferation and restrain cell apoptosis. *In vivo,* by inhibiting NO production, IGF-1 and IL-1Ra can promote cell proliferation and restrain catabolism of the cartilage matrix, thus inhibiting cartilage degeneration and increasing cartilage repair. Of course, in addition to their effects on NO production, IGF-1 and IL-Ra may promote cartilage repair in the other ways. There was no significant difference in the effect on inhibition of NO synthesis between IGF-1 and IL-Ra. However, IGF-1 increased the concentration of GAG, collagen II expression and cell proliferation to a greater extent than IL-1Ra *in vitro*; IL-1Ra decreased cell apoptosis *in vitro* and affected GAG concentrations *in vivo* to a greater extent than IGF-1. These results indicate that IGF-1 may promote the repair of cartilage defects mainly through the promotion of cartilage anabolism, while IL-Ra may act mainly through the inhibition of cartilage catabolism and degeneration to promote the repair of cartilage defects. Histological analysis showed that IGF-1 and IL-1Ra promoted a large amount of nascent cartilage located in the cartilage defect area. Safranin O staining and immunohistochemistry analysis indicated that the nascent cartilage had normally functioning chondrocytes synthesizing large amounts of proteoglycan and collagen II. These findings are similar to previously reported results [32], [37]. At the same time, in the IL-Ra group, nascent cartilage and inflammatory cell infiltration was significantly reduced compared to the IGF-1 group. When IGF-1 and IL-1Ra were co-expressed *in vitro*, the effects observed in the IGF-1 and IL-1Ra co-expression group were superior to those in the single-gene transfection groups by a number of measures, including the promotion of cell proliferation, the synthesis of GAG and collagen II, and the inhibition of apoptosis and NO synthesis in chondrocytes. *In vivo,* seven weeks after the introduction of the transgene, the concentrations of GAG and NO in the synovial fluid were lower than those of the single-gene transfection groups, and the expression of collagen II was higher than that of the single-gene transfection groups. A histological analysis indicated that the cartilage defects were completely filled with new cartilage cells, which could be similarly stained by Safranin O and immunohistochemistry in the same manner as the surrounding normal cartilage; Mankin score was significantly decreased; the numbers of inflammatory cells and fibroblasts were greatly reduced. These results suggest that the synergistic effects of IGF-1 and IL-Ra were obviously superior to the effects of IGF-1 or IL-Ra alone, not only for promoting cartilage proliferation but also for inhibiting the inflammatory response, chondrocyte apoptosis and cartilage degeneration.

In conclusion, as a non-viral vector, ^p^SP-CS can efficiently transfer an exogenous gene into rabbit chondrocytes both *in vitro* and *in vivo* and result in the efficient expression of the exogene. These results suggest that^ p^SP-CS is a good gene carrier and is a promising candidate for use in gene therapy for rabbit cartilage defects. Further work is necessary to study the ^p^SP-CS transfection efficiency in cells from other species, including human chondrocytes, and the synergistic beneficial effects of selected genes in cartilage defects.
